# Hand hygiene: dreams come true “Clean Care is Safer Care”

**DOI:** 10.1186/2047-2994-4-S1-P150

**Published:** 2015-06-16

**Authors:** C Hon Chung Radley, L Conita, W Isadora, KC H, L Shirley, L Annie, C Gloria, CC P, C Regina, Y Ida, C Queenie, C Patricia, C Christina

**Affiliations:** 1 Infection Control Team, Hospital Authority - Queen Marry Hospital, HKSAR, Hong Kong; 2 Hospital Authority, Hong Kong; 3 HK Sanatorium & Hospital, Hong Kong; 4 Baptist Hospital, Hong Kong

## Introduction

Hundreds of millions of patients around the world are affected by health care-associated infections (HCAIs) that are also the most frequent adverse event in healthcare system. Most are preventable through adherence to patient-care-practices. Hand hygiene is the most effective practice in preventing and controlling HCAI as well as community infections.

## Objectives

Clean Your Hands is a major component of the WHO First Global Patient Safety Challenge. It is a global campaign to improve hand hygiene among health-care workers. Since 2008 Hong Kong Infection Control Nurses’ Association (HKICNA) has been actively participating in the WHO Clean Hands Saves Lives campaign. It is proactively to promote hand hygiene in different healthcare settings and in the community.

## Methods

Since 2008 till now, during the World Health Carnival, HKICNA ran a booth to promote hand hygiene in the community. We were happy to find that there were over a thousand public to participate in hand hygiene education and games.

## Results

In 2012, HKICNA organized a poster design competition, the main theme was to promote hand hygiene in the hospital environment. The winner posters was used as talking wall in community and healthcare settings while others were used as design background of promotional gimmicks such as pen, tote bag etc. A hand-held electric fan was designed with visual lit up “hand hygiene" that again helps to remind healthcare workers the importance of hand hygiene.

In addition, two Hand Hygiene Dances were designed to continuously support and promote WHO's initiative on hand hygiene. The two Hand Hygiene Dances demonstrate hand hygiene should start from young children to adulthood, from healthcare worker to different professions in the community. Both versions are highly promoted in hospitals and schools in Hong Kong and assessable in YouTube gaining thousands of 'likes'.

## Conclusion

Hong Kong Infection Control Nurses' Association is fully committed in promoting infection prevention and control especially hand hygiene practices in healthcare and community. The endeavour of "Clean Hand Save Lives" will continue.

## Disclosure of interest

None declared.

